# Integrative Multi-Analysis Identifies METTL3-Regulated FGF19 and H6PD as Candidate Targets in Diabetic Cognitive Impairment

**DOI:** 10.3390/biom16030468

**Published:** 2026-03-20

**Authors:** Jun Fu, Huarui Wang, Junjie Yan, Weiyuan Chen, Ruguang Wang, Hongchang Gao, Chen Li

**Affiliations:** 1 State Key Laboratory of Macromolecular Drugs and Large-Scale Manufacturing, School of Pharmaceutical Sciences, Wenzhou Medical University, Wenzhou 325035, Chinawhr@wmu.edu.cn (H.W.);; 2 Innovation Academy of Testing Technology, Scientific Research Center, Oujiang Laboratory, Wenzhou Medical University, Wenzhou 325035, China; 3 Key Laboratory of Efficacy Evaluation of Traditional Chinese Medicine and Encephalopathy Research of Zhejiang Province, Wenzhou Medical University, Wenzhou 325035, China

**Keywords:** diabetic cognitive impairment, m^6^A methylation, METTL3, ^1^H-NMR metabolomics

## Abstract

Diabetic cognitive impairment (DCI) is a serious and growing public health concern. The role of N6-methyladenosine (m^6^A), the predominant mRNA modification in the mammalian brain, in DCI pathogenesis remains not fully elucidated. Here, GEO-derived diabetes datasets were combined with *in vivo* and *in vitro* models to reveal aberrant expression of m^6^A-related genes. The results showed that the overall level of m^6^A RNA methylation in both the diabetic group and the high-glucose group was significantly decreased compared to the normal group. In addition, the expression of methyltransferase METTL3, which is involved in the regulation of m^6^A RNA methylation, was downregulated in both diabetic and hyperglycemic groups, and was positively correlated with the downregulation of the overall m^6^A level. Neuronal models with stable METTL3 knockdown were generated using lentiviral transduction. Subsequent ^1^H-NMR metabolomic and MeRIP-qPCR analyses demonstrated that METTL3 deficiency disrupts key metabolic pathways, including phosphatidylethanolamine and phosphatidylcholine biosynthesis and glucose–alanine metabolism, and identified *Fgf15* (the mouse ortholog of human FGF19) and *H6PD* as candidate downstream targets. Collectively, these data suggest that METTL3-dependent m^6^A RNA methylation alterations may contribute to DCI through metabolic dysregulation, positioning METTL3 as a promising therapeutic target for DCI.

## 1. Introduction

Diabetes mellitus (DM) is a prevalent metabolic disease characterized by persistent hyperglycemia due to insulin resistance or impaired insulin secretion [1]. With the global population aging, the incidence of diabetes has significantly risen worldwide. According to the International Diabetes Federation (IDF), there are about 537 million people with diabetes worldwide in 2021, expected to increase to 783 million by 2045, affecting approximately one in eight adults [2]. Along with disturbed blood glucose regulation, diabetics suffer from a range of chronic complications, including retinopathy [3], renal failure [4], cardiovascular disease [5] and neuropathy [6]. Notably, diabetic cognitive impairment (DCI) is increasingly recognized as an important complication of diabetes and the severity of cognitive impairment is directly proportional to the progressive deterioration of diabetes [7,8,9]. Although the correlation between diabetes and cognitive dysfunction has been noted in epidemiological and clinical data and by researchers in various countries [10], who have put forward hypotheses regarding neuropathological mechanisms such as neuroinflammation, oxidative stress, mitochondrial dysfunction, and impaired insulin signaling [11,12,13], unfortunately, the pathological mechanisms of DCI remain incompletely elucidated.

With the development of sequencing technology in recent decades, epigenetics-related research has penetrated into multiple disciplines and fields, and has gained impressive achievements in understanding disease mechanisms and developing treatments [14,15,16]. As a complement to classical genetics, epigenetics research mainly focuses on DNA methylation, histone modification, chromatin remodeling, and other related aspects. N6-methyladenosine (m^6^A), as the most abundant RNA modification in eukaryotic cells, has been found to play a crucial role in regulating mRNA metabolism and various biological processes [17,18,19]. The methylation modification of m^6^A is a dynamic and reversible process that is governed by three key factors: methyltransferase ‘writers’, demethylase ‘erasers’, and methylation reading proteins ‘readers’. The intricate interplay among these components is essential for maintaining the intracellular m^6^A levels in a balanced state [20]. Typically, m^6^A RNA is modified by a core methyltransferase complex consisting of methyltransferase-like protein 3 (METTL3) and methyltransferase-like protein 14 (METTL14), while demethylation modification occurs through the action of demethylases FTO and ALKHB5 [21]. As the core protein of methyltransferase, METTL3 has been shown to play a non-negligible role in neurodegenerative diseases such as Alzheimer’s disease [22] and Parkinson’s disease [23]. Mechanistically, it has been shown that knockdown of the m^6^A methyltransferase METTL3 in macrophages attenuates the m^6^A modification of DNA methyltransferase 3A (*Dnmt3a*) mRNAs, which alleviates disease manifestations in mice with Alzheimer’s disease [24]. Additionally, METTL3 has been found to enhance long-term memory, and the knockdown of METTL3 leads to reduced m^6^A modification in mice, causing cognitive deficits [25], while overexpression of METTL3 rescues β-amyloid (Aβ)-induced synaptic damage and cognitive deficits *in vivo* [22].

Emerging evidence has established a critical link between dysregulated m^6^A modification and DCI. Recent studies have utilized diverse diabetic models to comprehensively profile m^6^A modifications in the hippocampus and explore the functional roles of specific m^6^A regulators. For example, m^6^A and RNA sequencing of the hippocampus from high-fat diet-induced DCI mice revealed differentially m^6^A-modified and expressed genes enriched in synaptic transmission and axonal guidance pathways, along with altered expression of the methyltransferases METTL3 and METTL14 and the demethylase FTO [26]. In STZ-induced type 1 diabetic mice, overexpression of the m^6^A reader YTHDF1 in the hippocampus was shown to ameliorate cognitive dysfunction, implicating YTHDF1 as a potential therapeutic target [27]. A comprehensive m^6^A sequencing analysis of the hippocampus from diabetic rats identified thousands of differentially methylated m^6^A peaks and differentially expressed genes, underscoring the widespread remodeling of the m^6^A landscape under diabetic conditions [28]. Moreover, a mechanistic study demonstrated that high glucose suppresses the demethylase ALKBH5, leading to increased m^6^A modification of *Dgkh* mRNA and subsequent tau hyperphosphorylation via PKC-α activation; notably, overexpression of *Dgkh* rescued tau pathology and cognitive deficits [21]. Collectively, these findings establish that m^6^A modification is altered in the diabetic hippocampus and that specific m^6^A regulators, including METTL3, YTHDF1, and ALKBH5, contribute to DCI pathogenesis through distinct downstream pathways. However, whether m^6^A dysregulation represents a common mechanism across type 1 and type 2 diabetes, how METTL3 deficiency affects neuronal metabolism, and which specific targets mediate its effects in DCI remain largely unexplored.

The aim of this study was to elucidate the role and underlying mechanism of METTL3-dependent m^6^A RNA methylation dysregulation in DCI. To this end, m^6^A methylation alterations were investigated in both *in vivo* and *in vitro* models of DCI, and METTL3-regulated metabolic pathways and downstream targets were explored. The results of this study may provide a new understanding of the role of m^6^A RNA methylation in DCI and will provide potential diagnostic predictors and therapeutic targets for DCI.

## 2. Materials and Methods

### 2.1. GEO Data Acquisition

The following datasets were downloaded from the Gene Expression Omnibus (GEO) database: GSE25724 (GPL96, [HG-U133A] Affymetrix Human Genome U133A Array [hermo Fisher Scientific, Santa Clara, CA, USA]) included 7 normal control (NC) and 6 diabetes mellitus (DM) islet samples; GSE156035 (GPL20844, Agilent-072363 SurePrint G3 Human GE v3 8x60 K Microarray 039494 [Agilent Technologies, Palo Alto, CA, USA]) included 20 NC and 20 DM peripheral blood mononuclear cell (PBMC) samples; GSE30528 (GPL571, [HG-U133A_2] Affymetrix Human Genome U133A 2.0 Array [hermo Fisher Scientific, Santa Clara, CA, USA]) included 13 NC and 9 diabetic nephropathy (DN) human glomerular samples; GSE34451 (GPL15011, Agilent-016475 Patkany genome 4x44 K [Agilent Technologies, Palo Alto, CA, USA]) included 3 NC and 6 DM rat striatum samples; GSE150489 (GPL21290, Illumina HiSeq 3000 [Illumina, San Diego, CA, USA], Homo sapiens) included 3 NC and 3 *si-METTL3* human HCC cell samples; GSE130012 (GPL21290, Illumina HiSeq 3000, *Homo sapiens*) included 3 wild-type (WT) and 3 METTL3-knockout (KO) human HCT116 cell samples; and GSE106613 (GPL17021, Illumina HiSeq 2500 [Illumina, San Diego, CA, USA], *Mus musculus*) included 3 WT and 3 *METTL3-KO* mouse embryonic stem cell (mESC) samples. To capture both peripheral and central transcriptional changes associated with diabetes, we included diverse diabetes-relevant tissues (islets, PBMCs, kidney, striatum) from human and rat. Cancer cell line datasets (GSE150489, GSE130012) were used for hypothesis generation due to the lack of public neuronal *METTL3* perturbation data; all candidates were subsequently validated in neuronal SH-SY5Y cells and diabetic mouse hippocampus. Gene annotation was performed in a Perl framework using the platform-specific annotation files for each dataset. For microarray datasets, background correction and between-array normalization were performed using the “normalizeBetweenArrays” function in the “limma” R package, and expression values were transformed using log2(TPM + 1) to facilitate downstream analyses and visualization.

### 2.2. The Exploration of m^6^A Expression Pattern

Twenty-three m^6^A regulators were examined, including six m^6^A writers (*METTL3*, *METTL14, WTAP, ZC3H13, RBM15B, *and *CBLL1*), two m^6^A erasers (*ALKBH5 *and *FTO*), and fifteen m^6^A readers (*YTHDC1, YTHDC2, YTHDF1, YTHDF2, YTHDF3, HNRNPC, FMR1, LRPPRC, HNRNPA2B1, IGFBP1, IGFBP2, IGFBP3, RBMX, ELAVL1, *and *IGF2BP1*). The expression levels of these m^6^A genes in the DM group and the normal group were visualized using boxplots. Differentially expressed m^6^A genes (*p* < 0.05) were identified using the ‘limma’ package of R software (4.2.1). Additionally, Pearson correlation analysis was conducted to assess the relationships among the seven differentially expressed m^6^A genes. To quantify the global m^6^A regulatory status of each sample, an m^6^A score was calculated based on the expression profiles of 23 m^6^A-related regulators (*METTL3, METTL14, WTAP, ZC3H13, RBM15B, CBLL1, ALKBH5, FTO, YTHDC1, YTHDC2, YTHDF1, YTHDF2, YTHDF3, HNRNPC, FMR1, LRPPRC, HNRNPA2B1, IGFBP1, IGFBP2, IGFBP3, RBMX, ELAVL1 *and *IGF2BP1*). Principal component analysis (PCA) was performed on the normalized expression matrix of these regulators across samples, and the first principal component (PC1) was extracted as the m^6^A score. Genes with *p* value < 0.05 and ∣log2FC∣ ≥ 0.25 without FDR were selected for clustering samples and constructing the m^6^A score. The m^6^A score is defined as m^6^Ascore = Σ(PC1i + PC2i), where i represents the expression levels of differentially expressed m^6^A regulators [29]. The m^6^A score reflects the global transcriptional activity of the m^6^A regulatory network rather than directly measuring m^6^A modification abundance. A higher score indicates greater overall expression of m^6^A-related genes, which may reflect tissue-specific regulatory states, but does not necessarily equate to the net biochemical outcome of m^6^A methylation.

### 2.3. Antibodies

The following antibodies were used in this study: anti-GAPDH (Affinity BiosciencesCincinnati, OH, USA) (1:1000), anti-m^6^A (Synaptic Systems, Göttingen, Germany, 202003) (for Dot blot:1:1000, for IHC: 1:500), anti-METTL3 (15073-1-AP, Proteintech, Rosemont, Illinois, USA) (for WB:1:2000, for IHC: 1:750), anti-METTL14 (HPA038002, Sigma-Aldrich, St. Louis, MO, USA) (for WB:1:2000, for IHC: 1:1000), anti-WTAP (60188-1-Ig, Proteintech) (for WB:1:2000, for IHC: 1:500), FTO (27226-1-AP, Proteintech) (for WB:1:1000, for IHC: 1:400), ALKBH5 (16837-1-AP, Proteintech) (for WB:1:2000, for IHC: 1:200), Goat Anti-Mouse IgG H&L (HRP, Affinity Biosciences) (1:10000), Goat Anti-Rabbit IgG H&L (HRP, Affinity Biosciences, Cincinnati, OH, USA) (1:10000), and Goat Anti-Rabbit IgG H&L (Alexa Fluor^®^ 488, Abcam, Cambridge, UK) (1:500).

### 2.4. Animals

Seven-week-old SPF-grade male C57BL/6 mice were obtained from Beijing Vital River Laboratory Animal Technology Co., Ltd. (Beijing, China) Male *db/db* (C57BLKS/J-*leprdb/leprdb*) mice and age-matched wild-type db/m (C57BLKS/J-*LepRdb/+*) mice were obtained from the Model Animal Research Center of Nanjing University. All experimental animals were housed in the SPF-grade animal room of the Animal Experiment Center at Wenzhou Medical University under standard conditions. After 1 week of acclimatization, C57BL/6 mice were randomly divided into two groups (*n* = 6 for each group). The mice in the model group underwent a 12 h fast followed by intraperitoneal injection of a freshly prepared 1% streptozotocin (STZ) solution at a dose of 50 mg/kg prepared in citrate buffer. Food was reintroduced 2 h after injection, and this procedure was repeated once daily for five consecutive days [30]. The fasting tail vein blood glucose levels were measured 3 days after the last injection to confirm successful diabetes induction in mice with blood glucose levels exceeding 11.1 mmol/L. The animals were euthanized 11 weeks after the onset of diabetes. Throughout the modeling and disease progression period, mice had ad libitum access to food and water. Prior to euthanasia, the weights of both groups of mice were recorded, and their blood glucose levels were monitored. Diabetic *db/db* mice and age-matched wild-type *db/m* mice were euthanized 10 weeks after diabetes onset following a 3-week stabilization period (*n* = 6 for each group). Before euthanasia, the weights of both groups were measured, and blood glucose levels were monitored. To ensure unbiased assessment, all investigators involved in outcome measurements (including blood glucose and body weight) were blinded to the group allocation throughout the study. All procedures were conducted in strict accordance with the Wenzhou Medical University Laboratory Animal Care and Use Manual (wydw-2020-0124).

### 2.5. Morris Water Maze (MWM) Test

Eleven weeks subsequent to STZ injection, the MWM test was employed to assess the learning and memory capabilities of the mice. Briefly, the initial four days were dedicated to the place navigation test, during which the escape latency of the mice to locate and ascend onto the concealed platform in the target quadrant from various quadrants was documented. On the fifth day, the concealed platform was removed for the spatial exploration test, and the trajectories of the mice and the frequency of their crossings of the target area were recorded [31,32,33]. The Viewer III software (Biobserve GmbH, Bonn, Germany) was utilized to analyze parameters such as the position, velocity, and movement trajectory of the mice.

### 2.6. Specimen Collection and Storage

Following the completion of the behavioral experiment, mice were anesthetized with isoflurane and then euthanized by cervical dislocation without perfusion. The brain was quickly removed, and the hippocampus and cortex tissues were separated and placed into frozen tubes, which were immediately frozen in liquid nitrogen and stored at −80 °C until use. Additionally, brain tissues from each group of three mice were fixed in 4% paraformaldehyde for pathological examination.

### 2.7. Quantitative Real-Time PCR

Total RNA was extracted using the Trizol reagent in accordance with the manufacturer’s protocol. Subsequently, the total RNA was reverse-transcribed into cDNA using HiScript III RT SuperMix(R323, Vazyme, Nanjing, China) for qPCR. For quantitative real-time PCR (qPCR), each reaction mixture (10 µL) contained 2 µL of cDNA, 0.5 µL each of forward and reverse primers (for target genes *METTL3, METTL14, WTAP, FTO, *and *ALKBH5*, as well as reference genes *GAPDH* and *β-ACTIN*), 5 µL of SYBR Green Master Mix, and 2 µL of RNase-free water. The qPCR amplification was performed using the ChamQ Universal SYBR Master Mix (Q711-02, Vazyme, Nanjing, Jiangsu, China) with the following thermal cycling conditions: initial denaturation at 95 °C for 2 min, followed by 40 cycles of 95 °C for 15 s and 60 °C for 45 s. A melting curve analysis was conducted according to the instrument’s default settings. The cycle threshold (Ct) values were recorded, and the relative mRNA expression levels of the target genes were calculated using the 2^−ΔΔCt^ method, with *GAPDH* and *β-ACTIN* serving as internal controls for normalization.

### 2.8. m^6^A Dot Blotting Assay

Dot blotting experiments were performed as previously reported [34]. Briefly, the total RNA (400 ng) was denatured at 70 °C for 5 min, cooled, and mixed with 20× SSC buffer, followed by transfer, UV crosslinking, and blocking. It was incubated overnight at 4 °C with anti-m^6^A antibody (1:1000, 202003, Synaptic Systems, Göttingen, Germany). After washing the membrane, it was incubated with the secondary antibody (Goat Anti-Rabbit IgG H&L, Affinity Biosciences) and the membrane was visualized using a chemiluminescence system. Methylene blue (MB) staining served as a control. To confirm signal specificity, a duplicate RNA sample was incubated with RNase A (100 µg/mL in PBS, 1 h at 37 °C) prior to spotting. The control resulted in no detectable signal, confirming the RNA dependence and antibody specificity of the detected m^6^A signals. Quantification of dot intensities was performed using ImageJ software (version 1.52a, NIH). The intensity of each m^6^A dot was normalized to the intensity of its corresponding methylene blue-stained dot to correct for RNA loading variations.

### 2.9. LC-MS/MS Assay for m^6^A Quantification

Poly(A) mRNA was extracted according to the instructions of the VAHTS mRNA Capture Beads Extraction Kit (Vazyme, Nanjing, Jiangsu, China), and the concentration of poly(A) mRNA was determined using a DeNoVIX Ultra-Micro Spectrophotometer (DeNovix, Wilmington, DE, USA). For each sample, 20 ng of poly(A) mRNA was digested with 1 µL S1 nuclease (180 U/µL), 2 µL alkaline phosphatase (1 U/µL), and the corresponding buffers in a total volume of 40 µL at 37 °C for 6 h. After digestion, samples were centrifuged at 12,000 g for 15 min at 4 °C, and the supernatant was analyzed by UPLC-MS/MS. Chromatographic separation was performed on a Waters ACQUITY UPLC BEH C18 column (2.1 × 100 mm, 1.7 µm) with a mobile phase consisting of water containing 0.02% formic acid (A) and methanol (B) at a flow rate of 0.4 mL/min. The gradient elution program was: 0–2.5 min, 4–31% B; 2.5–3.0 min, 31–69% B; 3.0–4.0 min, 69–95% B; 4.0–4.05 min, 95–4% B; and 4.05–6.0 min, 4% B. Mass spectrometric detection was performed on an API 6500 Q-TRAP (AB SCIEX) in the positive electrospray ionization mode with multiple reaction monitoring. The mass spectrometric parameters were as follows: curtain gas, nebulizer gas (GS1), and turbo-gas (GS2) were set at 30 psi, 55 psi, and 55 psi, respectively; the ion Spray Voltage was 5500 V; and source temperature 550 °C. The ion pairs of *m/z* 282.1/150.1 (Declustering Potential: 36 V, Collision Energy: 24 V) and *m/z* 282.1/123.1 were used for quantification [35]. Standard curves were constructed using reference substances over a concentration range of 0.01–500 ng, yielding the linear regression equation y = 45,203x − 15.838 with a correlation coefficient (R^2^) of 0.9999. The m^6^A content in each sample was calculated by interpolating the peak area from the calibration curve and normalized to the input amount of poly(A) mRNA.

### 2.10. Cell Culture

Human neuroblastoma SH-SY5Y cells were obtained from the American Type Culture Collection (ATCC CRL-2266) and maintained in DMEM/F12 (Dulbecco’s Modified Eagle’s Medium/Nutrient Mixture F12 Ham’s media, Biosharp, Hefei, Anhui, China ) medium. The medium was supplemented with 10% FBS serum, 100 U/mL penicillin and 0.1 mg/mL streptomycin and incubated in a cell culture incubator at 37 °C containing 5% CO_2_.

Cell viability was assessed using the CCK8 kit (Nanjing Jiancheng Bioengineering Institute, China). Cells were seeded in a 96-well plate at a density of 8 × 10^3^ cells/mL. Once the cells were firmly attached, they were treated with normal complete culture medium containing a gradient of high-glucose concentrations (0, 10, 25, 50, 75, 100, 125, 150 mM) for 24 h. To distinguish the specific effects of high glucose from potential hyperosmotic effects, an osmotic control experiment was performed. The results of this control experiment are presented in Supplementary Figure S1.

### 2.11. Cellular Immunofluorescence

Cells were inoculated in 24-well plates, washed with PBS, fixed with 4% paraformaldehyde at room temperature for 45 min. Cells were permeabilized with 0.5% Trition-X-100 solution and blocked with 5% BSA at room temperature for 60 min. The primary antibody was added and the solution was incubated. After washing with PBS, the secondary antibody was added and the solution was incubated in the dark for 30 min. The anti-fluorescence quencher was dropped on the glass slide, which was then covered and observed with a fluorescence microscope [36].

### 2.12. Cell Transfection

SH-SY5Y cells were transduced with lentiviruses encoding shRNA targeting METTL3 or a non-targeting control (functional titer ~1 × 10^8^ TU/mL) at MOI = 5 in the presence of polybrene (8 µg/mL) [37]. After 16 h, the medium was replaced with fresh complete medium. Puromycin selection (1 µg/mL) was initiated 24 h later and continued for 5 days until all non-transduced control cells were eliminated. The medium containing puromycin was refreshed every day. Surviving cells were expanded and used as stable *METTL3* knockdown lines. Knockdown efficiency was confirmed at both the mRNA and protein levels. *METTL3* mRNA levels were measured by RT–qPCR and normalized to *GAPDH* using the 2^−ΔΔCt^ method. Protein levels were assessed by immunoblotting using anti-METTL3 antibodies, with GAPDH as loading controls (Figure S2).

### 2.13. Western Blot

SH-SY5Y cells were lysed with RIPA buffer containing 1% phenylmethylsulfonyl fluoride, and proteins were extracted. Protein concentration was determined by BCA method. For Dot blot analysis, 400 ng of protein per sample was loaded directly onto a nitrocellulose membrane. For Western blot analysis, 30 μg of protein per sample was separated by sodium dodecyl sulfate–polyacrylamide gel electrophoresis (SDS-PAGE) and then transferred onto a PVDF membrane. The membrane was blocked with 5% skimmed milk for 60 min at room temperature and incubated overnight at 4 °C with primary antibody. Subsequently, the membrane was incubated with secondary antibody for 1 h. Finally, the membrane was visualized using an appropriate amount of ECL chemiluminescent solution.

### 2.14. ^1^H NMR-Based Metabolomics Analysis

Cells were digested with trypsin and centrifuged. Methanol and chloroform (v:v 2:1) were added to the precipitate, which was sonicated on ice for 30 min; an aqueous solution of 50% chloroform was then added and vortexed, and finally the supernatant was centrifuged. The supernatant was lyophilized and re-dissolved in D_2_O containing 0.08 mM TSP and centrifuged for NMR detection. The ^1^H-NMR spectrum was collected using a Bruker AVANCE III 600 MHz nuclear magnetic resonance spectrometer (Bruker, Billerica, MA, USA). The ZGPR pulse sequence was performed with the parameters of spectrum width = 12,000 Hz, acquisition time = 2.65 s/scan, number of data acquisition points = 32 K, and acquisition standard temperature = 298.0 K. Prior to Fourier transformation, the window function LB = 0.3 was added, and the FID signal was zeroed to 64 K. All acquired spectra were phase and baseline adjusted using Bruker Topspin 2.1 software. Metabolites were identified and assigned by comparing their spectral peaks with the Chenomx NMR Suite 7.5 database; only metabolites with unambiguous matching (match score > 0.90) were included in subsequent quantitative analysis. For multivariate statistical analysis, the spectral region from 0.0 to 9.0 ppm (excluding the water resonance region at 4.70–5.00 ppm) was segmented into bins of 0.01 ppm width to reduce data dimensionality while preserving metabolic pattern information. For precise quantification of individual metabolites, a finer integration interval of 0.0015 ppm was applied. To minimize inter-sample variation, metabolite concentrations were calculated as relative units (r.u.) based on the peak area normalized to the total spectral area of each sample. Samples were processed in randomized order during data acquisition to minimize systematic bias, and all analyses were performed within a single batch to avoid inter-batch variability. The processed dataset was imported into SIMCA-P+ 12.0 software for multivariate pattern recognition analysis. Orthogonal partial least squares discriminant analysis (OPLS-DA) was employed to identify metabolites contributing to group separation, with variable importance in projection (VIP) > 2 considered major contributors. Model robustness was evaluated using a 200-times permutation test with the R^2^Y and Q^2^ values indicating model fit and predictive capability.

For univariate analysis, Benjamini–Hochberg false discovery rate (FDR) correction was applied to all metabolite comparisons, with q < 0.05 considered significant. Only metabolites meeting both criteria (q < 0.05 and VIP > 2) were defined as robust differential metabolites. Pathway enrichment analysis was performed using MetaboAnalyst 5.0 with the hypergeometric test against the KEGG human pathway library; pathways with FDR-adjusted q < 0.05 were considered significantly enriched. Effect sizes (Cohen’s d) were calculated for key metabolites to assess biological significance.

### 2.15. MeRIP-qPCR

Changes in m^6^A methylation levels of downstream genes in sh-*METTL3* and OE-*METTL3* SH-SY5Y cells were validated using the EpiQuik CUT&RUN m^6^A RNA enrichment (MeRIP) kit (Epigentek, USA). Total RNA was extracted from sh-NC, sh-*METTL3*, and OE-*METTL3* SH-SY5Y cells. The RNA was then fragmented into 100–150 base pair segments. These fragments were subsequently immunoprecipitated using magnetic beads coated with approximately 2 µg of anti-m^6^A antibody. For each immunoprecipitated (IP) sample, a corresponding input sample (fragmented RNA before IP) was saved. Following washing and elution steps, the co-immunoprecipitated RNA was isolated. Both input and IP RNA were used as templates for subsequent RT-qPCR analysis. Primer sequences for *FGF19* and *H6PD* are listed in Supplementary Table S1. The enrichment of m^6^A-modified RNA was calculated using the formula 2^(Ct(Input)–Ct(IP))^, where Ct(Input) was adjusted for the dilution factor of the input aliquot. This normalization accounts for differences in basal expression levels of the genes.

### 2.16. Statistical Analysis

All statistical analyzes were completed using SPSS 13.0 (IBM Software, Chicago, IL, USA), and data results are expressed as mean ± SD. For multi-group comparisons, one-way ANOVA followed by Tukey’s HSD (all pairwise) post hoc tests was used. For comparisons between two groups, if both normality and equal variance assumptions were met (*p* > 0.05), statistical significance was determined using a two-tailed unpaired Student’s t-test. If either assumption was violated (*p* < 0.05), the non-parametric Mann–Whitney U test was employed instead. For transcriptomics and metabolomics data, Benjamini–Hochberg false discovery rate (FDR) correction was applied with q < 0.05 considered significant, metabolomics also required VIP > 2 from OPLS-DA analysis. A *p*-value of less than 0.05 was considered statistically significant.

## 3. Results

### 3.1. Expression Patterns of m^6^A Regulators in NC and DM Samples

**Figure 1:**
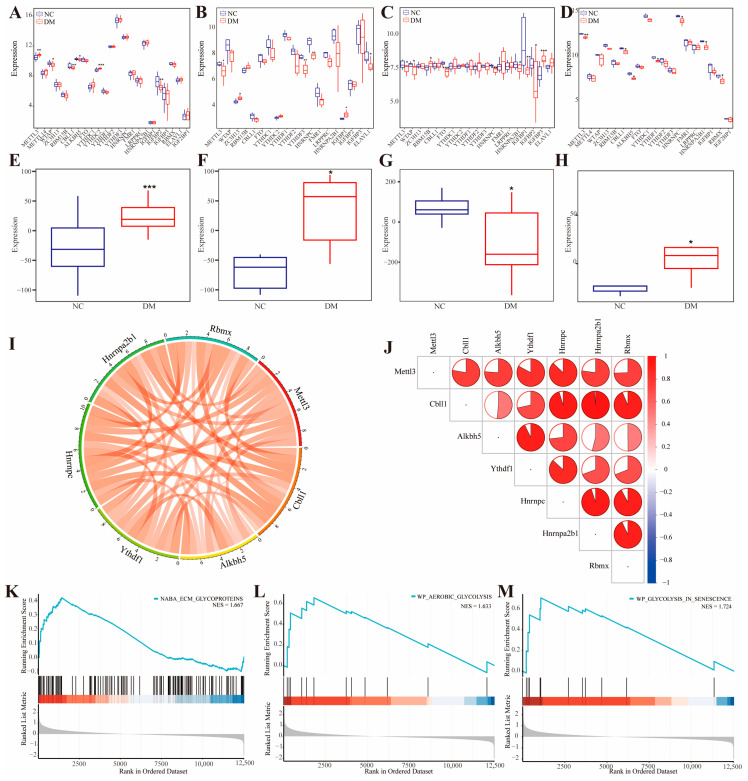
Expression patterns of m^6^A regulators in NC and DM samples. The scores of m^6^A regulators between NC and DM samples in peripheral blood (**A**), islet (**B**), kidney (**C**) and rat brain tissue (**D**). The m^6^A RNA methylation in peripheral blood (**E**), islet (**F**), kidney (**G**) and rat brain tissue (**H**) of the NC group and DM group. (**I**,**J**) The correlation among m^6^A regulators in DM samples via spearman correlation analysis. (**K**–**M**) Top three pathways are positively associated with the top seven pathways that are positively associated with METTL3. Data were analyzed using the independent sample *t*-test, * *p* < 0.05, ** *p* < 0.01, *** *p* < 0.001.

To elucidate the potential role of m^6^A-related genes in DM, differential expression analysis was conducted to investigate the expression patterns of these genes across various biological samples from both DM and NC groups. In human peripheral blood, *METTL3* and *YTHDC2* were significantly upregulated, whereas *WTAP, CBLL1, ALKBH5, *and *IGFBP2* were markedly downregulated in the DM group relative to the NC group (Figure 1A). Within pancreatic islets, *ZC3H13* and *IGFBP1* exhibited significant upregulation, while *METTL3, YTHDF3, HNRNPC, *and *ELAVL1* showed significant downregulation in the DM group compared to the NC group (Figure 1B). In kidney tissues, *RBM15B *and *IGFBP3* were significantly upregulated, whereas *METTL3, WTAP, FTO, HNRNPC, HNRNPA2B1, IGFBP1, *and *IGFBP2* were significantly downregulated in the DM group relative to the NC group (Figure 1C). Additionally, in rat brain tissue, a notable downregulation of *METTL3, CBLL1, ALKBH5, YTHDF1, HNRNPC, HNRNPA2B1, *and *RBMX* was observed in the DM group compared to the NC group (Figure 1D). Furthermore, PCA was employed to calculate the m^6^A scores of the corresponding samples. The results indicated that, except for kidney tissues, the m^6^A scores of the DM group were significantly higher than those of the NC group in the remaining biological samples (Figure 1E–H). Interestingly, this discrepancy in kidney tissue likely reflects the tissue-specific downregulation of multiple m^6^A regulators (including *METTL3, WTAP, FTO, HNRNPC, *and *IGFBPs*) observed in Figure 1C, highlighting the context-dependent nature of epitranscriptomic regulation in different diabetic target organs. Correlation analysis revealed a strong association among differentially expressed m^6^A regulators, particularly a robust positive correlation between *METTL3* and other regulators (Figure 1I,J). To further explore the biological functions and pathways associated with *METTL3*, single-gene Gene Set Enrichment Analysis (GSEA) was performed. The findings demonstrated that METTL3 is involved in aerobic glycolysis, ECM glycoproteins, and glycolysis in senescence pathways (Figure 1K–M).

### 3.2. Establishment of a Diabetes Model and Measurement of Body Weight, Blood Glucose Levels, and Learning and Memory Capabilities

**Figure 2:**
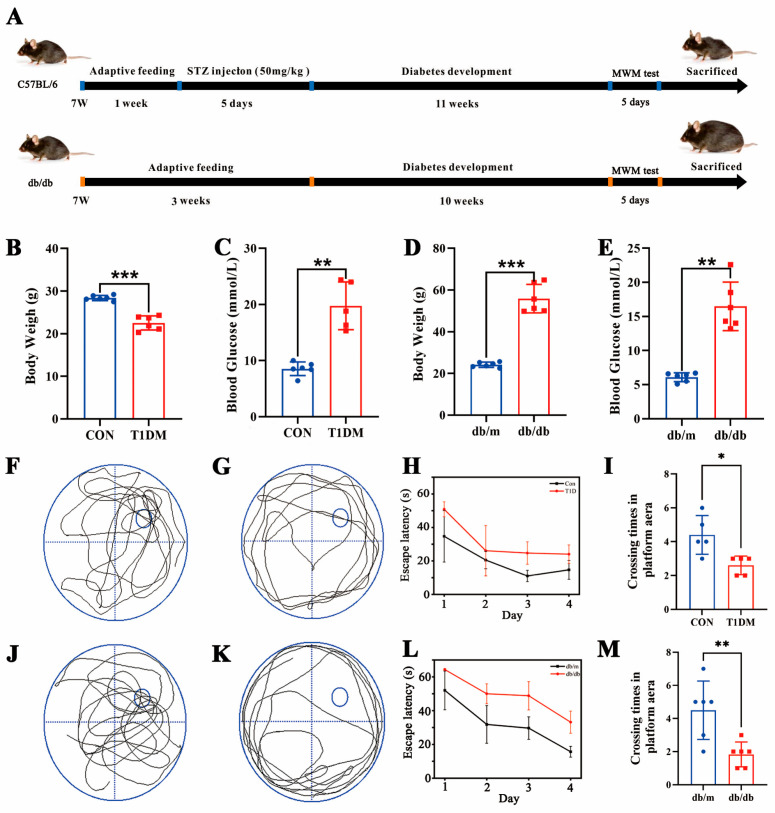
Changes in body weight and blood glucose during the development of diabetic animals. (**A**) Schematic diagram of disease progression in diabetic animals. (**B**) Body weight changes in T1D mice. (**C**) Blood glucose changes in T1D mice. (**D**) Body weight changes in db/db mice. (**E**) Body weight changes in db/db mice. Each group has *n* = 5–6 mice per group. The trajectory of Con (**F**) and T1D (**G**) mice in the space exploration experiment. (**H**) The escape latency of T1D and Con mice during the navigation period. (**I**) The crossing times of T1D and Con mice on target platforms in the space exploration test. The trajectory of *db/m* (**J**) and *db/db* (**K**) mice in the space exploration experiment. (**L**) The escape latency of db/m and db/db mice during the navigation period. (**M**) The crossing times of db/m and db/db mice on target platforms in the space exploration test. * *p* < 0.05, ** *p* < 0.01, *** *p* < 0.001 compared to the same experimental normal group.

The animal model of diabetic cognitive impairment was established according to the flowchart shown in Figure 2A. As shown in Figure 2B,C, both STZ-induced T1D mice and db/db mice showed a significant increase in blood glucose compared with the normal group and the blood glucose value was higher than 11.1 mmol/L, which proved that the diabetic animal model was successfully established. In addition, the T1D mice showed significant weight loss compared with the control group (Con, Figure 2D), while the db/db mice showed significant weight gain as the disease progressed (Figure 2E). The learning and memory capabilities of T1D mice were assessed through the MWM test (Figure 2F,G). Throughout the place navigation phase, the escape latency of both the T1D group and the Con group declined as the training time increased, and the T1D group exhibited longer escape latency compared to the Con group across training days (Figure 2H). On the fifth day of the spatial exploration test, the frequency of crossings of the T1D group was significantly lower than that of the Con group, suggesting that the learning and memory capabilities of the T1D group were significantly compromised (Figure 2I). Similarly, *db/db* mice exhibited significant cognitive impairment in the MWM test, with prolonged escape latency during the place navigation phase and reduced platform crossings in the spatial probe trial compared to *db/m* controls (Figure 2J–M). These results confirm that both type 1 and type 2 diabetic models develop DCI.

### 3.3. Decreased m^6^A RNA Modifications in the Hippocampus and Cortex of T1D Mice

**Figure 3:**
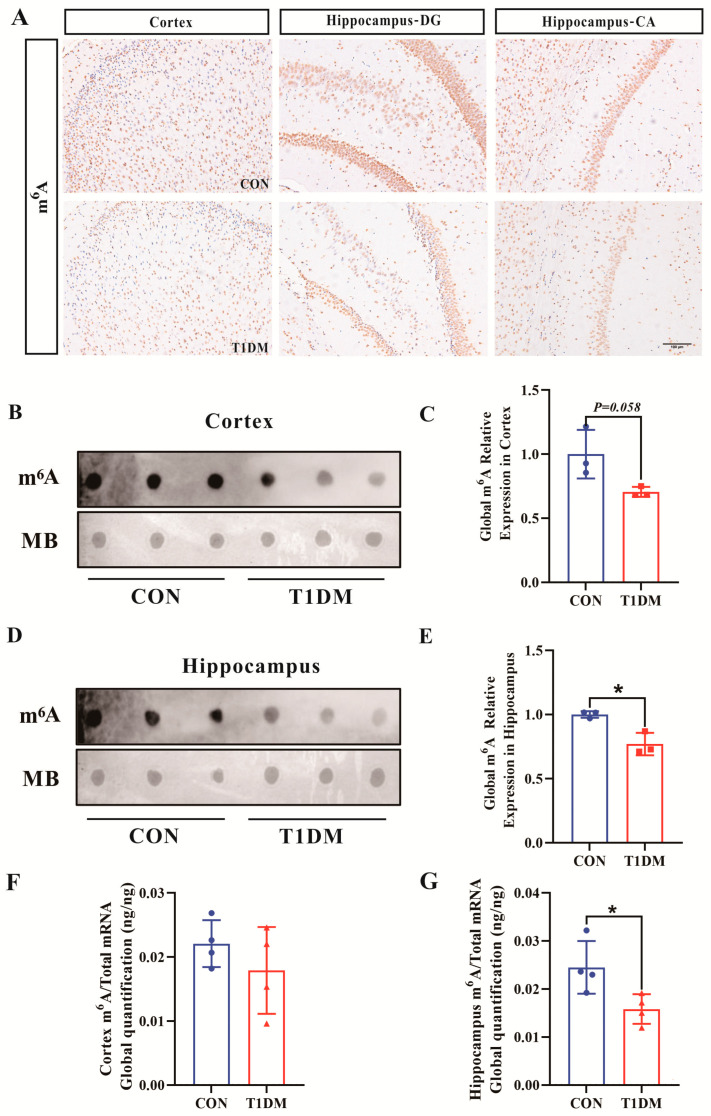
Decreased m^6^A RNA methylation levels in the cortex and hippocampus of T1D mice. (**A**) Immunohistochemistry of m^6^A in cortex and hippocampus (200×). Scale bar = 100 μm. (**B**) Dot blot analysis of m^6^A in cortex. (**C**) Relative quantification of cortical m^6^A Dot blot results. (**D**) Dot blot analysis of m^6^A in hippocampus. (**E**) Relative quantification of hippocampal m^6^A Dot blot results. (**F**) LC–MS/MS analysis of m^6^A levels in cortex. (**G**) LC–MS/MS analysis of m^6^A levels in hippocampus. Each group has *n* = 3–4 mice per group. * *p* < 0.05 compared with Con group.

To examine the alterations in m^6^A RNA methylation modification within DCI animal models, this study assessed the changes in m^6^A RNA methylation levels in the hippocampus and cortex of T1D mice. Immunohistochemical results showed that the m^6^A-positive spots in the DG and CA regions of the hippocampus and cortex in the T1D mice were significantly weakened compared with the Con group, indicating that the overall m^6^A RNA methylation level was decreased in the T1D mice (Figure 3A). Subsequently, m^6^A-specific dot blotting also confirmed this result. As shown in Figure 3B,D, the overall m^6^A RNA methylation level in the cortex of T1D mice decreased but not statistically significantly, while the hippocampal tissue showed a significant decrease (Figure 3C,E). Consistently, LC-MS/MS results also further confirmed the decrease in m^6^A RNA methylation levels in T1D mice (Figure 3F,G).

### 3.4. Decreased m^6^A RNA-Related Regulator Proteins in the Hippocampus and Cortex of T1D Mice

**Figure 4:**
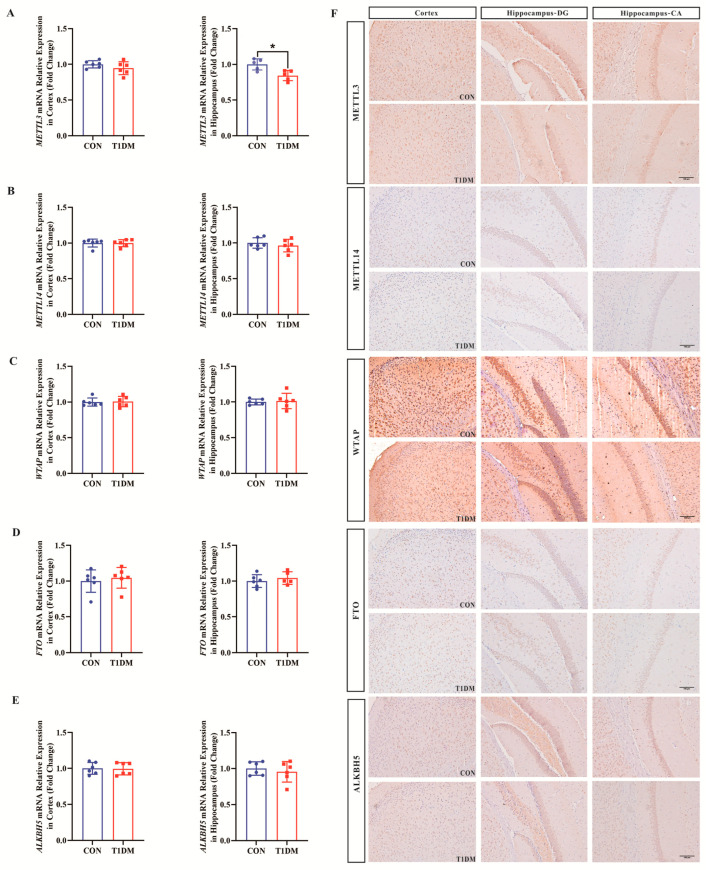
mRNA and protein levels of m^6^A RNA methylation-related enzymes in the cortex and hippocampus of T1D mice. (**A**) Relative quantification of METTL3 mRNA levels in cortex and hippocampus. (**B**) Relative quantification of METTL14 mRNA levels in cortex and hippocampus. (**C**) Relative quantification of WTAP mRNA levels in cortex and hippocampus. (**D**) Relative quantification of FTO mRNA levels in cortex and hippocampus. (**E**) Relative quantification of ALKBH5 mRNA levels in cortex and hippocampus. *n* = 5–6 mice per group. (**F**) Immunohistochemistry (200×) of METTL3, METTL14, WTAP, FTO, and ALKBH5 in cortex and hippocampus. Scale bar = 100 μm. *n* = 3 mice per group. Compared with the Con group, * *p* < 0.05.

To further investigate the mechanism by which the overall level of m^6^A RNA methylation decreased in the brain of T1D mice, we used RT-qPCR and immunohistochemistry to detect the mRNA and protein levels of key enzymes involved in the regulation of m^6^A RNA methylation, respectively. The results showed that the mRNA level and protein level of METTL3 in the hippocampal tissues of T1D mice were significantly decreased compared with those of the Con group, whereas there was no significant difference in the cortex (Figure 4A). However, there were no statistically significant differences in the mRNA levels and protein levels (Figure 4F) of other key enzymes involved in the regulation of m^6^A RNA methylation such as METTL14, WTAP, FTO, and ALKBH5 (Figure 4B–E). Therefore, it was hypothesized that the downregulation of METTL3 might be responsible for the overall decrease in the level of m^6^A RNA methylation in the brains of T1D mice.

### 3.5. Decreased m^6^A RNA Modifications in the Hippocampus and Cortex of db/db Mice

**Figure 5:**
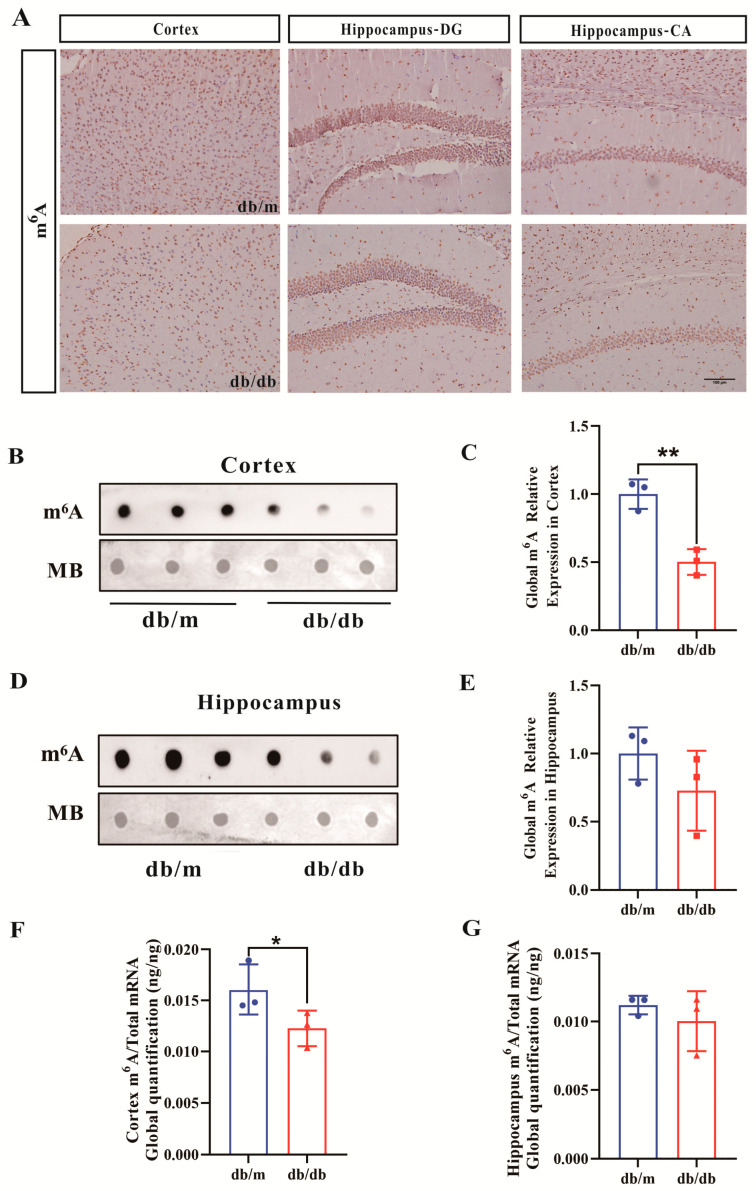
Decreased m^6^A RNA methylation levels in the cortex and hippocampus of db/db mice. (**A**) Immunohistochemistry of m^6^A in cortex and hippocampus (200×). Scale bar = 100 μm. (**B**) Dot blot analysis of m^6^A in cortex. (**C**) Relative quantification of cortical m^6^A Dot blot results. (**D**) Dot blot analysis of m^6^A in hippocampus. (**E**) Relative quantification of hippocampal m^6^A Dot blot results. (**F**) LC–MS/MS analysis of m^6^A levels in cortex. (**G**) LC–MS/MS analysis of m^6^A levels in hippocampus. For immunohistochemistry (**A**), *n* = 3 mice per group. For Dot blot and LC-MS/MS (**B**–**G**), *n* = 3–4 mice per group. * *p* < 0.05, ** *p* < 0.01 compared with Con group.

To further confirm the potential role of m^6^A RNA modification in DCI, we also examined the changes in m^6^A RNA methylation modification in db/db mice. Consistent with the above observation, the m^6^A-positive spots in the DG and CA areas of the hippocampus and cortex of db/db mice were also attenuated in comparison with the control group (Figure 5A). The m^6^A-specific Dot blot results of cortex (Figure 5B) and hippocampal (Figure 5D) tissues and the quantitative results of corresponding tissues (Figure 5C,E) found that the overall m^6^A RNA methylation level of cortical tissue in the db/db group significantly decreased, whereas hippocampal tissues tended to decrease but were not statistically different. The results of m^6^A-targeted mass spectrometry of cortex (Figure 5F) and hippocampal (Figure 5G) tissues were also consistent with the spot blotting results. In summary, it showed that the overall level of m^6^A RNA methylation also decreased in the brains of mice in the db/db group.

### 3.6. Decreased m^6^A RNA-Related Regulator Proteins in the Hippocampus and Cortex of db/db Mice

**Figure 6:**
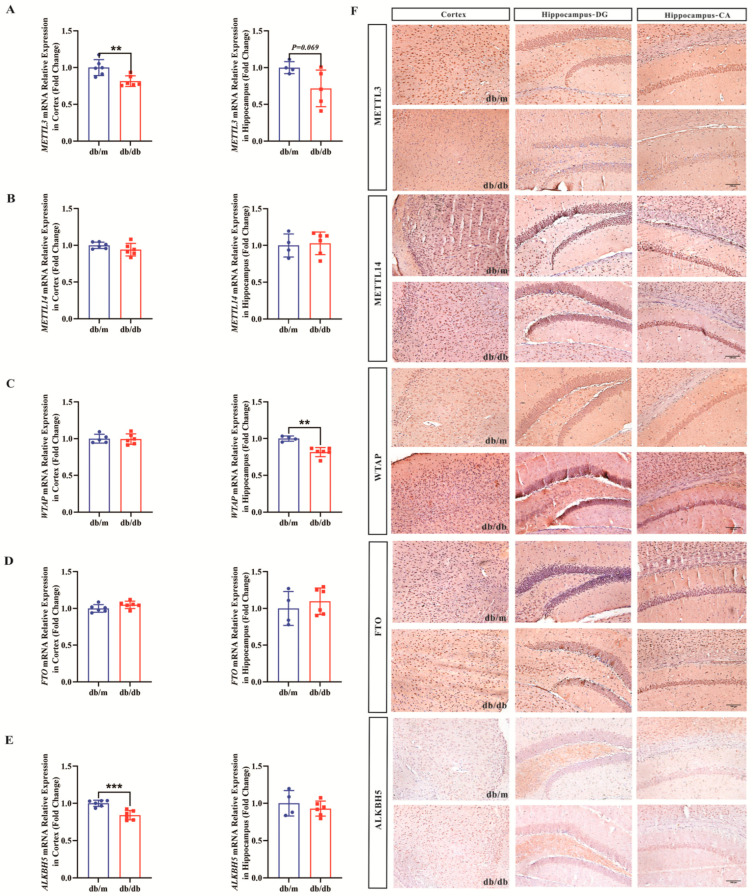
mRNA and protein levels of m^6^A RNA methylation-related enzymes in the cortex and hippocampus of db/db mice. (**A**) Relative quantification of METTL3 mRNA levels in cortex and hippocampus. (**B**) Relative quantification of METTL14 mRNA levels in cortex and hippocampus. (**C**) Relative quantification of WTAP mRNA levels in cortex and hippocampus. (**D**) Relative quantification of FTO mRNA levels in cortex and hippocampus. (**E**) Relative quantification of ALKBH5 mRNA levels in cortex and hippocampus. Each group above has *n* = 5–6 mice per group. (**F**) Immunohistochemistry (200×) of METTL3, METTL14, WTAP, FTO, and ALKBH5 in cortex and hippocampus. Scale bar = 100 μm. *n* = 3 mice per group. Compared with the Con group, ** *p* < 0.01, *** *p* < 0.001.

Similarly, mRNA levels and protein levels of key enzymes involved in m^6^A RNA methylation in the cortex and hippocampal tissues of *db/db* mice were examined using RT-qPCR and immunohistochemistry, respectively. The results showed that the mRNA levels and protein levels of METTL3 in both the cortex and hippocampal tissues of the *db/db* group were significantly decreased compared to those of the db/m group (as shown in Figure 6A). mRNA levels and protein levels of WTAP were also decreased in the hippocampal tissues of the *db/db* group, whereas it was not detected in cortex. The mRNA level and protein level of ALKBH5 were also found to be significantly decreased in the cortical tissues of the *db/db* mice, whereas no significant difference was detected in the hippocampal tissues. While the mRNA levels (Figure 6B,D) and protein levels (Figure 6F) of METTL14 and FTO were not statistically different in both cortical and hippocampal tissues between the two groups. Therefore, we speculate that the downregulation of METTL3, WTAP and ALKBH5 may have contributed to the decrease in the overall level of m^6^A RNA methylation in the brain of db/db mice.

### 3.7. Decreased m^6^A RNA Modifications in the High-Glucose-Induced SH-SY5Y Cells

**Figure 7:**
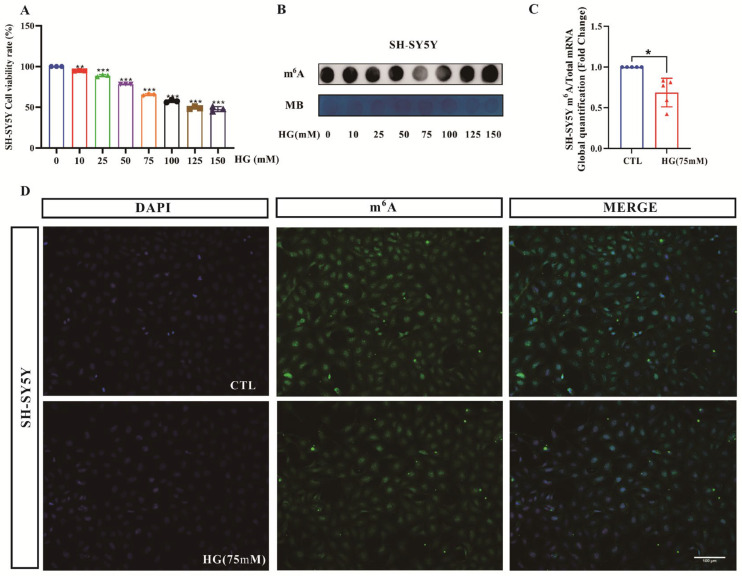
Changes in SH-SY5Y cell viability and m^6^A RNA methylation levels. (**A**) CCK8 assay to detect the effect of high sugar on SH-SY5Y cell viability. (**B**) Dot blot analysis of the overall level of m^6^A RNA methylation in cells under high-glucose gradient. (**C**) LC–MS/MS analysis of m^6^A levels induced by 75 mM high glucose. (**D**) 75 mM high-glucose-induced cellular immunofluorescence (200×). Scale bar = 100 μm. CTL: normal untreated group; HG (75 mM): 75mM high-glucose-treated group. For all assays, *n* = 3–4 independent biological replicates. Compared with the CTL group, * *p* < 0.05, ** *p* < 0.01, *** *p* < 0.001.

To investigate the cellular level changes in m^6^A RNA modification, we used an *in vitro* model of SH-SY5Y induced by high glucose. The results of the CCK8 assay showed that the cell viability of SH-SY5Y showed a significant concentration-dependent decrease under high-glucose induction (Figure 7A). Combined with the m^6^A-specific spot blotting results (Figure 7B), a concentration of 75 mM was selected as the optimal concentration to induce neuronal damage in SH-SY5Y. Then we used targeted mass spectrometry and immunofluorescence to detect the m^6^A RNA modification changes in SH-SY5Y cells induced by high glucose. Intriguingly, we found that the overall level of m^6^A modification *in vitro* was consistent with the results of the *in vivo*. The relative quantitative results of m^6^A targeted mass spectrometry showed that the levels of m^6^A in the high-glucose-induced group was significantly reduced compared with that in the normal group (Figure 7C). The results of cell immunofluorescence were the same as those of targeted mass spectrometry, and it was found that the fluorescence intensity of cells in the high-glucose-induced group was significantly weaker than that in the normal group (Figure 7D). In summary, the overall level of m^6^A was similarly decreased in high-glucose-induced SH-SY5Y cells.

### 3.8. Decreased m^6^A RNA-Related Regulators in the High-Glucose-Induced SH-SY5Y Cells

**Figure 8:**
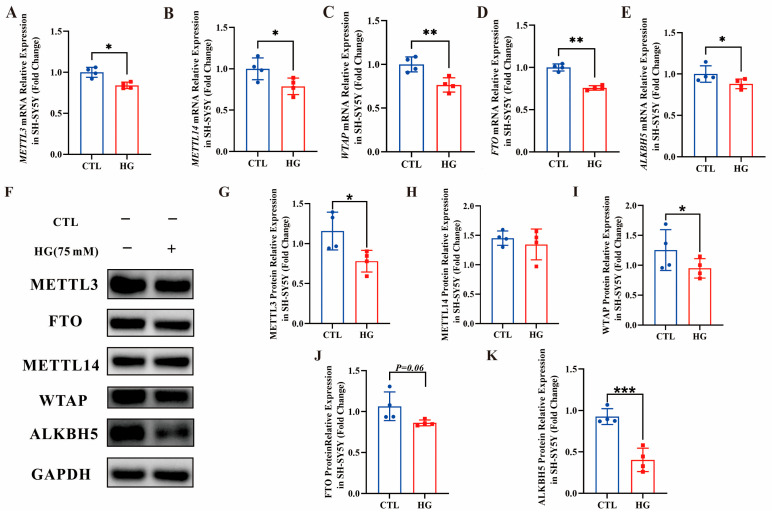
Expression of mRNA and protein levels of m^6^A RNA methylation-related enzymes in SH-SY5Y cells induced by 75 mM high glucose. (**A**) Relative quantification of METTL3 mRNA levels. (**B**) Relative quantification of METTL14 mRNA levels. (**C**) Relative quantification of WTAP mRNA levels. (**D**) Relative quantification of FTO mRNA levels. (**E**) Relative quantification of ALKBH5 mRNA levels. (**F**) Western blot of METTL3, METTL14, WTAP, FTO, and ALKBH5 protein levels. Original western blots can be found at Supplementary Materials. (**G**–**K**) Relative quantification of METTL3, METTL14, WTAP, FTO, and ALKBH5 protein levels. ctl: normal untreated group; HG (75 mM): 75 mM high-glucose-treated group. *n* = 4 independent biological replicates. * *p* < 0.05, ** *p* < 0.01, *** *p* < 0.001 compared with CTL group.

To further explore the potential mechanism of m^6^A RNA modification in high-glucose-induced SH-SY5Y cells, mRNA levels and protein levels of key enzymes involved in m^6^A RNA methylation were detected by RT-qPCR and Western blot analysis, respectively. As shown in Figure 8A–E, the expression of *METTL3, METTL14, WTAP, FTO *and *ALKBH5* at the mRNA level was significantly reduced compared with the normal group. The WB results (Figure 8F) and the corresponding relative quantification results (Figure 8G–K) showed that the expression of METTL3, WTAP, FTO and ALKBH5 in the high-glucose-induced group also decreased compared with the normal group. However, there was no statistical difference in the protein levels of METTL14 between the two groups, indicating that high-glucose induction may affect the transcription level of METTL14 but not its translation level.

### 3.9. METTL3 Knockdown Altered the Metabolic Profile of SH-SY5Y Cells

**Figure 9:**
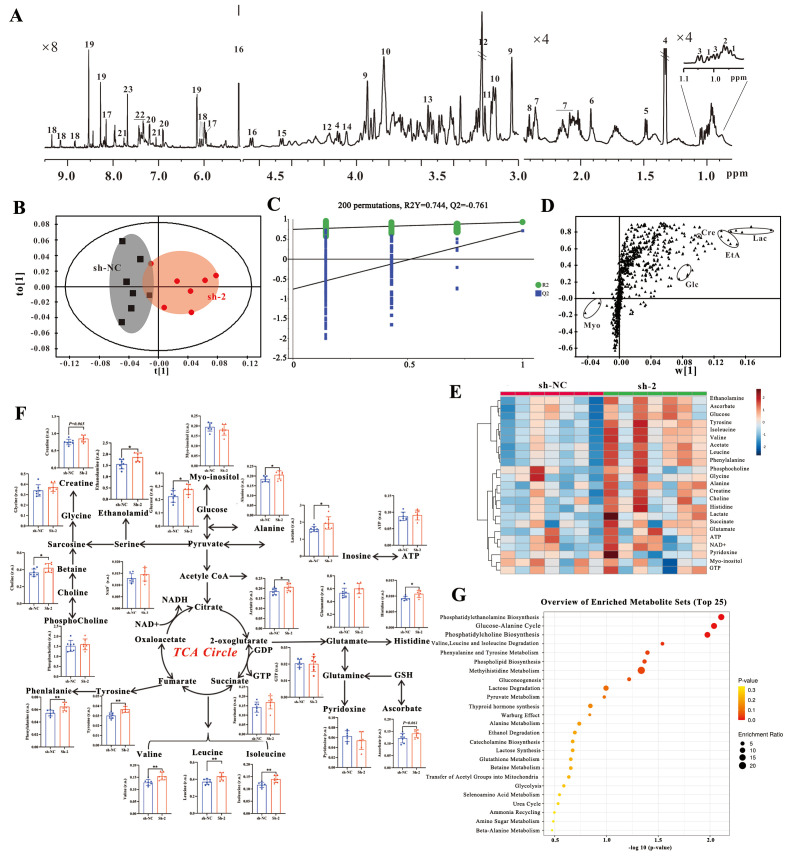
^1^H-NMR spectra and metabolic pattern recognition analysis of SH-SY5Y cells with METTL3 knockdown. (**A**) ^1^H-NMR spectra of metabolites in transfected SH-SY5Y cells. (**B**) OPLS-DA analysis of blank sh-NC group and knockdown sh-2 group. (**C**) Two hundred permutations of the OPLS-DA. (**D**) S-plot diagram of intracellular metabolites in the blank sh-NC group and knockdown sh-2 group. (**E**) Heat map of relative quantitative changes in intracellular metabolites in the blank sh-NC group and knockdown sh-2 group. (**F**) Relative quantitative metabolic pathway diagram of intracellular metabolites in the blank sh-NC group and knockdown sh-2 group. (**G**) Metabolite metabolic pathway enrichment diagram with statistically significant differences in cells between the blank sh-NC group and the knockdown sh-2 group. sh-NC: transfected blank control group; sh-2: transfected shRNA-METTL3-2 knockdown group. The number of samples in each group is *n* = 7. Compared with sh-NC group, * *p* < 0.05, ** *p* < 0.01.

Collectively, we found that m^6^A RNA methylation modification was decreased in both *in vivo* and *in vitro* models of DCI. Notably, METTL3, a key enzyme regulated by m^6^A RNA methylation, changed consistently in the *in vivo* and *in vitro* levels. Therefore, we constructed a METTL3 knockdown (sh-*METTL3*) SH-SY5Y cell model by the lentiviral transfection technique. To explore whether METTL3 affects metabolic changes and is involved in the mechanism of DCI, we conducted ^1^H-NMR metabolomics studies on cell samples from the blank group and the METTL3 knockdown group. The ^1^H-NMR spectrum of intracellular metabolites is shown in Figure 9A, and 23 metabolites were attributed, mainly including energy metabolism-related metabolites, neurotransmitter-related metabolites, amino acid metabolism-related metabolites, etc. To further confirm the differential contribution of metabolites, we used the OPLS-DA model (Figure 9B) to compare the metabolic patterns of the blank sh-NC group and the sh-2 group with the highest knockdown efficiency. The results of a 200-permutation test indicated that the OPLS-DA model was not overfitted and the results were reliable (Figure 9C). The corresponding S-plot is shown in Figure 9D. Combined with the results of heat map analysis (Figure 9E), it was found that the relative quantitative values of ethanolamine, glucose, lactic acid, histidine, acetic acid, alanine, leucine, isoleucine, valine, tyrosine, phenylalanine and choline in the tricarboxylic acid cycle pathway increased significantly in the knockdown sh-2 group compared with the blank sh-NC group. For other metabolites, there was no statistically significant difference between the two groups (Figure 9F). Then metabolites with statistical differences were analyzed for enrichment of metabolic pathways, and the results showed that metabolites with statistical differences between the two groups were mainly involved in the phosphatidylethanolamine biosynthesis pathway, glucose–alanine pathway, phosphatidylcholine biosynthesis pathway, etc. (Figure 9G).

### 3.10. The Exploration of Metabolism-Related Downstream Genes Regulated by METTL3

**Figure 10:**
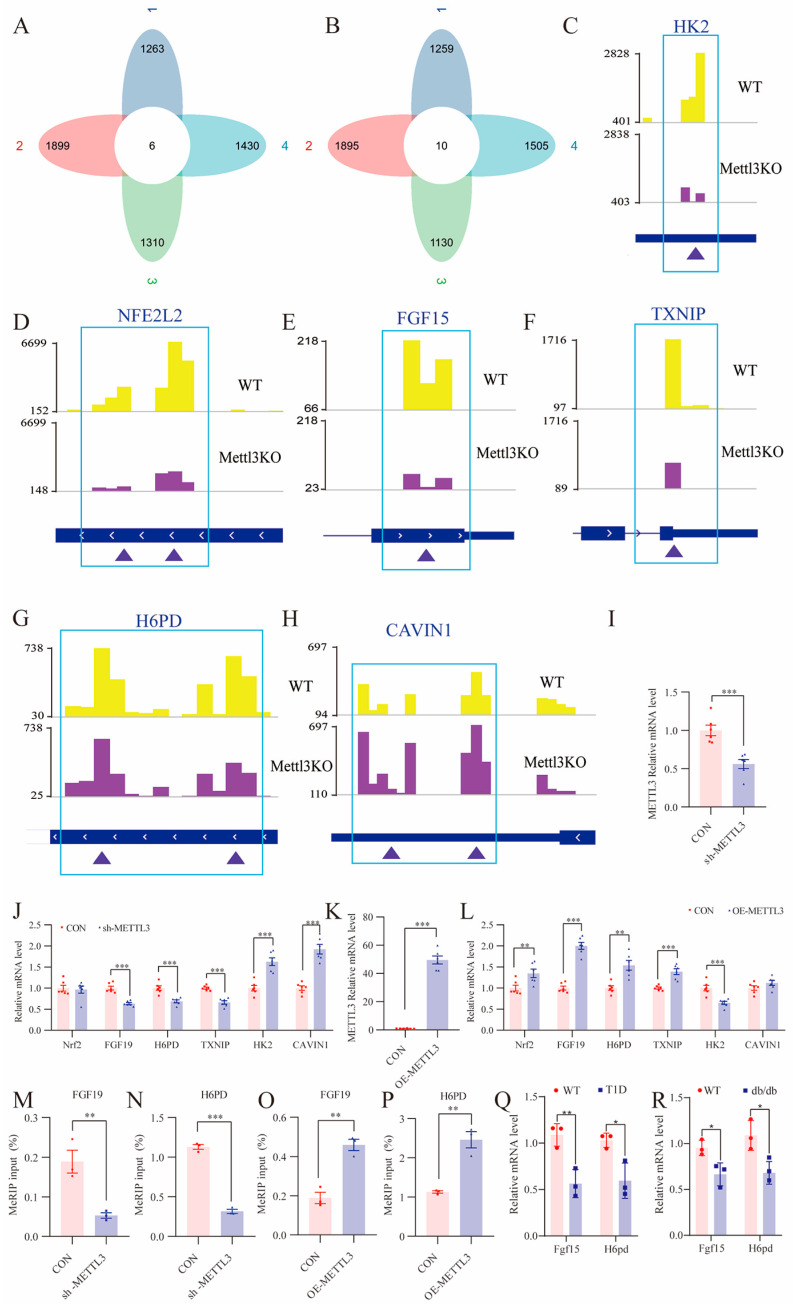
The exploration of metabolism-related downstream genes regulated by METTL3. The petal diagram displays the overlapping downregulated (**A**) and upregulated (**B**) genes in the diabetes-related, metabolism-related, and two sh-*METTL3* databases. (**C**–**H**) IGV plots showing examples of m^6^A peaks from GSE 106613. (**I**) qRT-PCR of the expression level of *METTL3* in sh-*METTL3* SH-SY5Y cells (*n* = 6 independent biological replicates). (**J**) qRT-PCR of the expression level of downstream genes in sh-*METTL3* SH-SY5Y cells (*n* = 6 independent biological replicates). (**K**) qRT-PCR of the expression level of METTL3 in OE-*METTL3* SH-SY5Y cells (*n* = 6 independent biological replicates). (**L**) qRT-PCR of the expression level of downstream genes in OE-*METTL3* SH-SY5Y cells (*n* = 6 independent biological replicates). (**M**,**N**) MeRIP-qPCR analysis detected the m^6^A modification levels in the sh-*METTL3* SH-SY5Y (*n* = 3 independent biological replicates). (**O**,**P**) MeRIP-qPCR analysis detected the m6 A modification levels in the OE-*METTL3* SH-SY5Y (*n* = 3 independent biological replicates). (**Q**) qRT-PCR of the expression level of *Fgf15 *and *H6PD* in WT and T1D mice (*n* = 3 mice per group). (**R**) qRT-PCR of the expression level of Fgf15 and H6PD in WT and db/db mice (*n* = 3 mice per group). * *p* < 0.05, ** *p* < 0.01, *** *p* < 0.001.

To identify metabolism-related downstream genes regulated by *METTL3* in diabetes models, a systematic multi-step filtering strategy was employed. First, diabetes-related genes were retrieved from the GeneCards database with a relevance score > 7, and metabolism-related genes were retrieved with a relevance score > 4. These were intersected with differentially expressed genes from two public *METTL3*-knockdown transcriptome datasets (GSE150489 and GSE130012), yielding six downregulated and ten upregulated genes that were both diabetes/metabolism-associated and responsive to *METTL3* loss (Figure 10A,B). Second, the m^6^A expression peaks of downstream genes in the GSE106613 m^6^A RIP-seq database were then analyzed using IGV and six genes that followed the trend observed in the petal plots were identified (Figure 10C–H). qRT-PCR validation confirmed that METTL3 mRNA levels were significantly reduced in sh-*METTL3* SH-SY5Y cells, while they were significantly increased in OE-*METTL3* SH-SY5Y cells (Figure 10I,K). Among the six candidates, *FGF19, H6PD, *and *TXNIP* showed significant downregulation in sh-*METTL3* cells, whereas *HK2 *and *CAVIN1* were significantly upregulated (Figure 10J). In OE-*METTL3* cells, *HK2* was significantly reduced, while *Nrf2, FGF19, H6PD,* and *TXNIP* were significantly elevated (Figure 10L). To further elucidate the downstream genes regulated by METTL3, gene-specific MeRIP-qPCR was used to detect m^6^A modification levels in sh-*METTL3* and OE-*METTL3* SH-SY5Y cells. The results showed that *FGF19* and *H6PD* exhibited the most robust and reproducible changes in m^6^A modification levels, with significant decreases in sh-*METTL3* cells and significant increases in OE-*METTL3* cells (Figure 10M–P). Collectively, these findings identify *FGF19* and *H6PD* as candidate downstream targets of METTL3-mediated m^6^A modification. To determine whether these *in vitro* findings translate to the *in vivo* context, we measured the expression of *FGF19 *and *H6PD* in hippocampal tissues from both diabetic mouse models. Notably, as mice do not possess the *FGF19* gene, we measured its functional ortholog, *Fgf15*. qRT-PCR analysis revealed that *Fgf15* mRNA levels were significantly reduced in the hippocampus of both STZ-induced T1D mice and *db/db* mice compared to their respective controls (Figure 10Q,R). Similarly, *H6PD* mRNA levels were significantly decreased in both diabetic models.

## 4. Discussion

DCI, as a complication of diabetes mellitus in the central system, seriously affects the quality of life of patients. Epidemiological studies have confirmed that the high prevalence of DCI will seriously challenge global public health systems and impose a heavy economic and social burden [38,39]. Therefore, there is an urgent need to understand the underlying mechanisms of DCI to provide new therapeutic strategies. In the present study, we demonstrated that m^6^A RNA methylation modification was significantly decreased in both *in vivo* and *in vitro* models of DCI and was mainly attributed to the downregulation of *METTL3*. In addition, *METTL3* knockdown changes the metabolic pattern of SH-SY5Y cells. The pathway enrichment results showed that the differential metabolites were mainly involved in the phosphatidylethanolamine biosynthesis pathway, the glucose–alanine pathway, and the phosphatidylcholine biosynthesis pathway.

According to our previous studies and literature reports, both STZ-induced T1D mice and db/db mice will develop cognitive dysfunction such as impaired learning and memory when diabetes develops to a certain period [40,41,42,43]. As a neuronal cell line that has been widely used in cognition-related central brain diseases, we used high-glucose-induced SH-SY5Y cells in this study to establish an *in vitro* neuronal damage model of DCI. Therefore, in order to better understand the potential pathogenesis of DCI, a variety of *in vivo* and *in vitro* models were combined in this study. An important physiological distinction between the two diabetic models used in this study merits discussion. Consistent with the characteristics of type 1 diabetes, STZ-induced mice exhibited significant weight loss, resulting from insulin deficiency-induced catabolism, muscle wasting, and adipose tissue breakdown. In contrast, *db/db* mice, a model of type 2 diabetes, displayed progressive weight gain driven by leptin receptor deficiency, hyperphagia, and insulin resistance. Despite these divergent metabolic trajectories, both models developed cognitive impairment, as demonstrated in our previous studies [41,42,43]. This dissociation between body weight and cognitive outcomes suggests that the pathogenesis of DCI is not merely a secondary consequence of obesity or weight loss, but rather is driven by factors common to both diabetic states. Chronic hyperglycemia, the shared feature of both models, likely serves as the primary driver of neurotoxicity through mechanisms such as oxidative stress, neuroinflammation, and mitochondrial dysfunction. These findings underscore that DCI can arise in diverse metabolic contexts, including both lean and obese phenotypes, thereby highlighting the central role of hyperglycemia-induced neuronal injury over systemic body weight changes. This observation also reinforces the translational relevance of our study, as DCI afflicts both type 1 and type 2 diabetic patients irrespective of their body habitus.

As the most abundant reversible mRNA modification in the brain, m^6^A has been shown to be associated with progressive neurological disorders, but the role of m^6^A RNA methylation modification in DCI remains unclear. In the present study, we found that the overall level of m^6^A RNA methylation in the brains of STZ-induced T1D mice and db/db mice decreased compared with the normal group. Consistent with the changes *in vivo*, the overall m^6^A RNA methylation at the SH-SY5Y cell level induced by high glucose *in vitro* was also attenuated, which was consistent with the results reported in the literature [44,45]. The formation of m^6^A is a dynamic and reversible process, mainly regulated by methyltransferases and demethylases. In this study, we found that the expression of *METTL3*, a key methyltransferase involved in the regulation of m^6^A RNA methylation, were all significantly decreased and positively correlated with the downregulation of overall m^6^A levels. However, there was no significant difference in the expression of demethylase *FTO* *in vivo*, and it was only significantly decreased *in vitro*. Interestingly, we observed a tissue-specific regulatory pattern of *METTL3* under diabetic conditions. While *METTL3* was downregulated in the brain, pancreatic islets, and kidney of diabetic rodents, it was significantly upregulated in PBMCs from diabetic patients. Instead of regarding this as a contradiction, we interpret this dichotomy as evidence of the context-dependent and tissue-specific nature of epitranscriptomic regulation in response to systemic metabolic stress. In PBMCs, which are directly exposed to circulatory inflammatory stimuli, *METTL3* upregulation may be part of an acute stress response or immune activation program. In contrast, the consistent downregulation of *METTL3* in insulin-sensitive tissues and the brain may represent a maladaptive consequence of chronic glucotoxicity and oxidative stress, potentially contributing to organ dysfunction. This tissue-specific pattern highlights that the role of *METTL3* in DCI is not uniform but rather context-dependent. Furthermore, the concordant dysregulation of *METTL3* observed across human clinical samples (PBMCs) and rodent target tissues (brain, islets) warrants discussion regarding cross-species validity. The m^6^A modification machinery, particularly the *METTL3* core component, is evolutionarily highly conserved between humans and rodents. Given that the STZ-induced rat model faithfully recapitulates many features of human diabetic encephalopathy [40,41], our cross-species comparison provides a valuable translational bridge. It suggests that the epigenetic mechanisms involving *METTL3* are fundamentally conserved and that findings from rodent models may offer relevant insights into human DCI pathology.

As a classic methylase, *METTL3* has been shown to be involved in the development of a variety of neurological diseases [46,47,48]. Zhang et al. found that *METTL3*-mediated m^6^A RNA methylation modification can enhance long-term memory [25]. Another study showed that acute knockdown of the hippocampal *METTL3* gene significantly impaired the spatial learning and memory abilities of mice [49]. Lentiviral infection mediates the ectopic expression of *METTL3* in the hippocampus of mice, and also causes obvious spatial cognitive deficits [50]. However, *METTL3* overexpression can rescue Aβ-induced synaptic damage and cognitive impairment *in vivo* [22]. In addition, *METTL3* deletion also inhibit neuronal development, thereby affecting the morphological maturation of new neurons in the adult brain [51]. The above results also further suggest that *METTL3* may be a potential therapeutic target for DCI, but the specific mechanism of *METTL3* still needs further exploration.

Given that m^6^A methylation is the most abundant modification in mRNA, it is involved in almost all stages of the RNA cycle [52]. The dynamic regulation of m^6^A RNA methylation changes the expression of signaling molecules and metabolic pathway-related genes, greatly affecting systemic metabolism and exhibiting different physiological functions [53,54]. Therefore, based on ^1^H-NMR metabolomics technology, this study found that *METTL3* knockdown SH-SY5Y cells exhibited different metabolic patterns. Among them, glucose, lactate, creatine, ethanolamine, and phosphocholine were the main contributing metabolites that change the intracellular metabolic pattern. Glucose and lactate act as key regulators of energy homeostasis in the brain, and glucose in the cells can be metabolized to pyruvate, which in turn enters the tricarboxylic acid cycle and is converted to lactate [55]. When diabetic cognitive dysfunction occurs, specific metabolic changes will occur in different brain regions. There was evidence that glucose and lactate will be significantly increased in the diabetic cognitive dysfunction group [40,41]. Zhao et al. reported that lactic acid accumulation in the brain causes cognitive decline in diabetic rats through the *GPR81*-dependent PKA-CREB signaling pathway [56]. In the present study, we similarly found that the knockdown of *METTL3* in neuronal cells significantly increased the intracellular metabolic content of glucose and lactate, suggesting that disruption of glucose and lactate metabolism due to the knockdown of *METTL3* in neuronal cells may be a causative factor in triggering cognitive dysfunction in diabetes.

To further clarify the downstream genes regulated by *METTL3*, MeRIP-qPCR was used to detect the m^6^A modification levels of the downstream genes. The results indicated that both *FGF19* and *H6PD* were significantly downregulated in sh-*METTL3* cells, while they were markedly upregulated in OE-*METTL3* SH-SY5Y cells. Research has demonstrated that *FGF19* functions as a hormone with pleiotropic metabolic effects, influencing insulin sensitivity, glycolipid metabolism, and energy balance. *FGF19* has been shown to enhance glucose metabolism in diabetic rodents and plays a crucial role in early neuronal development [57,58,59]. Given that diabetes is an independent risk factor for cognitive impairment, alterations in *FGF19* levels may impact cognitive function in diabetic patients. We hypothesize that *FGF19* may be implicated in the pathogenesis of DCI through *METTL3*-mediated RNA methylation. Additionally, *H6PD*, a key enzyme in glucose metabolism and the antioxidant defense system, has also been associated with DCI. Studies have revealed that *H6PD* is an essential component of the intracellular glucocorticoid-activated system and participates in the pathological processes of insulin resistance and metabolic syndrome [60,61].

The integration of our metabolomics findings with the identification of *METTL3* downstream targets provides a comprehensive mechanistic framework for understanding DCI pathogenesis. Our ^1^H-NMR metabolomics analysis revealed that *METTL3* knockdown in SH-SY5Y cells induced significant metabolic reprogramming, with glucose, lactate, creatine, ethanolamine, and phosphocholine identified as the key contributing metabolites distinguishing knockdown cells from controls. Quantitative analysis further demonstrated significant elevations in glucose, lactate, and multiple amino acids (including alanine, leucine, isoleucine, valine, tyrosine, and phenylalanine) in *METTL3*-deficient cells. Pathway enrichment analysis implicated the phosphatidylethanolamine biosynthesis pathway, glucose–alanine pathway, and phosphatidylcholine biosynthesis pathway as the primary metabolic routes affected by *METTL3* loss.

The accumulation of glucose and lactate in *METTL3*-deficient neuronal cells is particularly significant and can be mechanistically linked to *H6PD,* one of the downstream targets we identified. H6PD encodes hexose-6-phosphate dehydrogenase, a key enzyme in the pentose phosphate pathway that regulates glucose flux and maintains intracellular redox balance through NADPH generation. The *METTL3*-mediated reduction in *H6PD* m^6^A methylation and subsequent downregulation likely disrupts pentose phosphate pathway activity, shunting glucose toward glycolysis and thereby increasing lactate production. This metabolic shift explains the elevated lactate levels observed in our metabolomics data and has direct implications for cognitive function. Lactate activates *GPR81*, a G-protein-coupled receptor, which inhibits adenylate cyclase, reduces cAMP levels, and subsequently suppresses PKA-CREB signaling, a pathway essential for synaptic plasticity, long-term potentiation, and memory formation. In parallel, the observed alterations in ethanolamine and phosphocholine point to disrupted phospholipid metabolism, which may be linked to *FGF19*, another *METTL3* downstream target. The *METTL3*-mediated reduction in *FGF19* expression could contribute to impaired phospholipid homeostasis, affecting membrane integrity and synaptic function. Given that phosphatidylethanolamine and phosphatidylcholine are major components of neuronal membranes, their dysregulation may further compromise cognitive function.

Thus, we propose an integrated model wherein *METTL3* downregulation in DCI leads to reduced m^6^A methylation of *H6PD* and *FGF19* mRNAs. Reduced *H6PD* expression disrupts pentose phosphate pathway flux, causing glucose accumulation, shunting toward glycolysis, and elevating lactate. Increased lactate activates neuronal GPR81, suppressing PKA-CREB signaling and impairing synaptic plasticity. Concurrently, reduced *FGF19* expression disrupts phospholipid metabolism, compromising neuronal membrane integrity. These convergent pathways, including energy metabolism dysregulation, redox imbalance, and membrane phospholipid disruption, collectively contribute to the cognitive deficits observed in DCI.

While this study provides the integrated analysis linking *METTL3*-mediated m^6^A modification to metabolic dysregulation in DCI, several limitations should be acknowledged. First, the *in vivo* data presented herein are primarily associative: we observed reduced global m^6^A levels and *METTL3* expression in the brains of diabetic mice, and these changes coincided with cognitive impairment. Although we have now demonstrated that *Fgf15* (the mouse ortholog of human *FGF19*) and *H6PD* are significantly downregulated in the hippocampus of both STZ-induced T1D and *db/db* mice, and that their expression correlates positively with *METTL3* levels, these findings still represent correlational evidence rather than proof of causality. To ascertain that the absence of *METTL3* in the brain directly results in the onset of DCI, future studies should employ *METTL3*-specific knockdown or overexpression in diabetic animals, followed by comprehensive behavioral and metabolic assessments, which will be of great significance. Second, although our MeRIP-qPCR data demonstrate that *METTL3* perturbation alters m^6^A modification levels on *FGF19 *and *H6PD* transcripts, functional validation through luciferase reporter assays with m^6^A site mutagenesis is needed to confirm that these sites directly regulate mRNA stability or translation efficiency. Third, the proposed model wherein *H6PD* downregulation drives lactate accumulation via pentose phosphate pathway diversion, and wherein elevated lactate impairs cognitive function through GPR81-PKA-CREB signaling, remains hypothetical. Direct pharmacological or genetic modulation of *GPR81* in *METTL3*-manipulated neuronal cells or animal models would be required to establish this causal chain. Fourth, while our immunohistochemistry data demonstrate reduced METTL3 protein in the diabetic brain, this approach is semi-quantitative. Future studies should employ quantitative Western blot analysis with densitometry across biological replicates to precisely determine the magnitude of METTL3 reduction and to enable per-animal correlation analysis with global m^6^A levels measured by LC-MS/MS. Such analysis would provide direct evidence linking *METTL3* abundance to global m^6^A status in the diabetic brain. Additionally, the concurrent dysregulation of other m^6^A regulators (e.g., *WTAP*, *ALKBH5*) observed in specific regions of db/db mice suggests that the epitranscriptomic landscape in DCI likely involves multiple regulators; future studies should explore potential synergistic or compensatory interactions among these enzymes. Finally, while our cross-species comparison between human PBMCs and rodent tissues is informative, validation in human brain tissue, though challenging, would substantially strengthen the translational relevance of our findings. Future studies addressing these limitations will be essential to establish METTL3 and its downstream targets as viable therapeutic strategies for DCI.

## 5. Conclusions

In summary, we have confirmed the potential mechanism of *METTL3*-dependent dysregulation of m^6^A RNA methylation in DCI with *in vivo* data obtained from a variety of mouse models and *in vitro* data using a combination of pathophysiology, molecular biology, analytical chemistry and metabolomics. The findings indicated that differential metabolites were primarily associated with phosphatidylethanolamine biosynthesis, glucose–alanine pathways, and phosphatidylcholine biosynthesis, with *Fgf15* (the mouse ortholog of human *FGF19*) and *H6PD* emerging as potential downstream targets of *METTL3*-mediated m^6^A modifications. Collectively, the present study elucidated the pivotal role of *METTL3*-mediated m^6^A modification in DCI, and these findings are expected to provide novel insights that will enhance our understanding of DCI.

## Data Availability

All of the data is contained within the article and the Supplementary Materials.

## Supplementary Materials

The following supporting information can be downloaded at: https://www.mdpi.com/article/10.3390/biom16030468/s1, Figure S1: Mannitol control for hyperosmolarity effects. Figure S2: Validation of METTL3 knockdown efficiency. Table S1: Primers. Table S2: METTL3 shRNA sequences. Table S3: Quantitative densitometry data for Western blots.

## Abbreviations

The following abbreviations are used in this manuscript:

DM	Diabetes mellitus
IDF	International Diabetes Federation
DCI	Diabetic cognitive impairment
GEO	Gene Expression Omnibus
m^6^A	N6-methyladenosine
PCA	Principal component analysis
METTL3	Methyltransferase-like protein 3
METTL14	Methyltransferase-like protein 14
Dnmt3a	DNA methyltransferase 3A
DN	Diabetic nephropathy
PBMC	Peripheral blood mononuclear cell
Con	Control group
WT	Wild-type
MWM	Morris water maze
qPCR	Quantitative real-time PCR
GSEA	Gene Set Enrichment Analysis
