# Supplementary material for: Integrative Multi-Analysis Identifies METTL3-Regulated FGF19 and H6PD as Candidate Targets in Diabetic Cognitive Impairment
Source: Biomolecules. 2026 Mar 20;16(3):468. doi: 10.3390/biom16030468 (PMC13023568; doi:10.3390/biom16030468)
Supplement: Supplementary file 1 [file biomolecules-16-00468-s001.zip › biomolecules-4152481-supplementary.pdf]

Article

Integrative Multi-Analysis Identifies METTL3-Regulated FGF19 and H6PD as Candidate Targets in Diabetic Cognitive Impairment

Jun Fu [1,2,3,†], Huarui Wang [1,†], Junjie Yan [1], Weiyuan Chen [1], Ruguang Wang [1], Hongchang Gao [1,2,3,*] and Chen Li [1,2,3,*]

1 State Key Laboratory of Macromolecular Drugs and Large-Scale Manufacturing, School of Pharmaceutical Sciences, Wenzhou Medical University, Wenzhou 325035, China
2 Innovation Academy of Testing Technology, Scientific Research Center, Oujiang Laboratory, Wenzhou Medical University, Wenzhou 325035, China
3 Key Laboratory of Efficacy Evaluation of Traditional Chinese Medicine and Encephalopathy Research of Zhejiang Province, Wenzhou Medical University, Wenzhou 325035, China
* Correspondence: gaohc27@wmu.edu.cn (H.G.); lichen@wmu.edu.cn (C.L.)
† These authors contributed equally to this work.

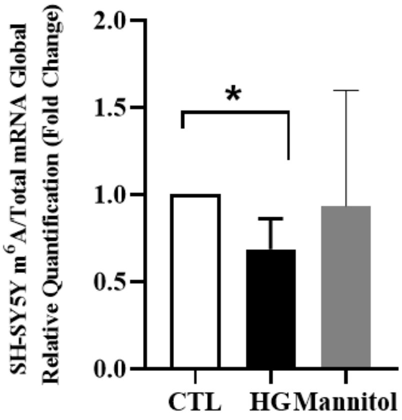

Figure S1. Mannitol control for hyperosmolarity effects. SH-SY5Y cells were treated with normal glucose, high glucose, or equimolar mannitol for 24 h. m^6A levels were measured by LC-MS/MS. Data are presented as mean ± SEM (n=3 per group). *$p < 0.05$ vs. normal glucose control

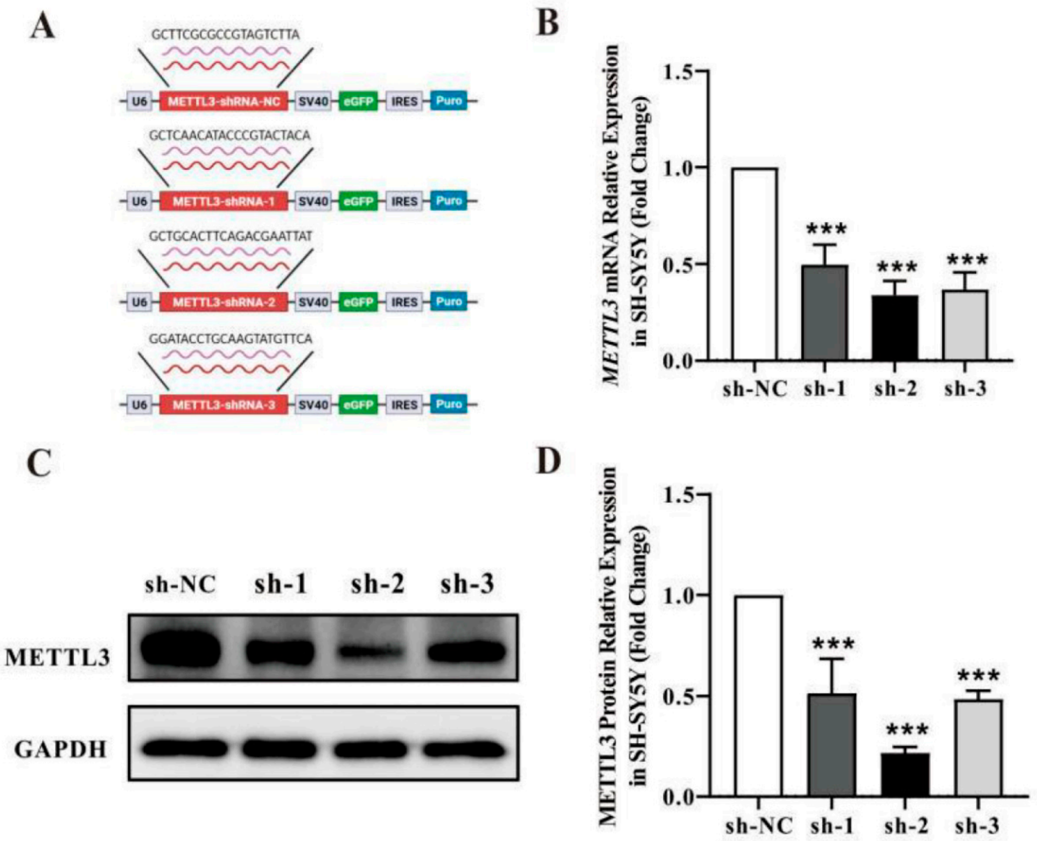

Figure S2. Validation results of METTL3 knockdown efficiency in SH-SY5Y cells. (A) Schematic diagram of METTL3 blank and knockdown sequence fragments. (B) Expression of mRNA levels in SH-SY5Y cells knockdown of METTL3. (C) Expression of protein level in SH-SY5Y cell knockdown of METTL3. (D) Relative quantification of protein expression in SH-SY5Y cells knockdown of METTL3. sh-NC: transfected blank control group; sh-1: transfected shRNA-METTL3-1 knockdown group; sh-2: transfected shRNA-METTL3-2 knockdown group; sh-3: transfected shRNA-METTL3-3 knockdown group. Number of samples per group n=4. $^{***}P<0.001$ compared with sh-NC group.

Table S1. List of primers used in this study

Primer name	Source	Primer sequence (5' to 3')
GAPDH-F	M	5’-AGGTCGGTGTGAACGGATTTG-3’
GAPDH-R	M	5’-TGTAGACCATGTAGTTGAGGTCA-3’
METTL3-F	M	5’-TTAGCATCTGGTCTGGCCTCTT-3’
METTL3-R	M	5’-TGACCTTCTTGCTCTGCTGTTC-3’
METTL14-F	M	5’-GACTGGCATCACTGCGAATGA-3’
METTL14-R	M	5’-AGGTCCAATCCTTCCCCAGAA-3’
WTAP-F	M	5’-TGCACGCAGGGAGAACATTC-3’
WTAP-R	M	5’-TGAACTTGCTTGAGGTACTGGAT-3’
FTO-F	M	5’-GACACTTGGCTTCCTTACCTG-3’
FTO-R	M	5’-CTCACCACGTCCCGAAACAA-3’
ALKBH5-F	M	5’-CGCGGTCATCAACGACTACC-3’
ALKBH5-R	M	5’-ATGGGCTTGAACTGGAACTTG-3’
β-ACTIN-F	H	5’-TTCTACAATGAGCTGCGTGTG-3’
β-ACTIN-R	H	5’-GGGGTGTTGAAGGTCTCAAA-3’
METTL3-F	H	5’-CAAGCTGCACTTCAGACGAA-3’
METTL3-R	H	5’-GCTTGGCGTGTGGTCTTT-3’
METTL14-F	H	5’-GAGTGTGTTTACGAAAATGGGGT-3’
METTL14-R	H	5’-CCGTCTGTGCTACGCTTCA-3’
WTAP-F	H	5’-GGCAGAGGAGGTAGTGGTTA-3’
WTAP-R	H	5’-TTTGATTGGTGTGTGAGAGAGTTT-3’
FTO-F	H	5’-GAAGGCTAATGAGGATGCTGTG-3’
FTO-R	H	5’-GTTGTATGCTGCTCTGCTCTT-3’
ALKBH5-F	H	5’-TCATCAACGACTACCAGCC-3’
ALKBH5-R	H	5’-GAAGGACACGGACACGAT-3’
NFE2L2-F	H	5’-TCCAAGTCCAGAAGCCAAACTGAC-3’
NFE2L2-R	H	5’-GGAGAGGATGCTGCTGAAGGAATC-3’
TXNIP-F	H	5’-AGCCAGCCAACTCAAGAGACAAAG-3’
TXNIP-R	H	5’-AGACAGACACCCGCCCATCAG-3’
H6PD-F	H	5’-GAAAGAGACCGTGGATGCTGAAGG-3’
H6PD-R	H	5’-GCCTCCGCACTGCTGACATTG-3’
HK2-F	H	5’-CGTGCCCGCCAGAAGACATTAG-3’
HK2-R	H	5’-CTTGCTCAGACCTCGCTCCATTTC-3’
CAVIN1-F	H	5’-ACGAGGCGGTGGAGGTTGAG-3’
CAVIN1-R	H	5’-CGCACCTTGGTCTTCTCCATCTTC-3’
FGF19-F	H	5’-GGCTTTCTTCCACTCTCTCATTTCC-3’
FGF19-R	H	5’-GGTCCATGCTGTCGGTCTCC-3’

Table S2. The sequence of *METTL3* shRNA used to construct a lentiviral vector

Name	Primer sequence (5' to 3')
shRNA-NC	GCTTCGCGCCGTAGTCTTA
shRNA-METTL3-1	GCTCAACATACCCGTACTACA
shRNA-METTL3-2	GCTGCACTTCAGACGAATTAT
shRNA-METTL3-3	GGATACCTGCAAGTATGTTCA

Extended Data of Figure. 8

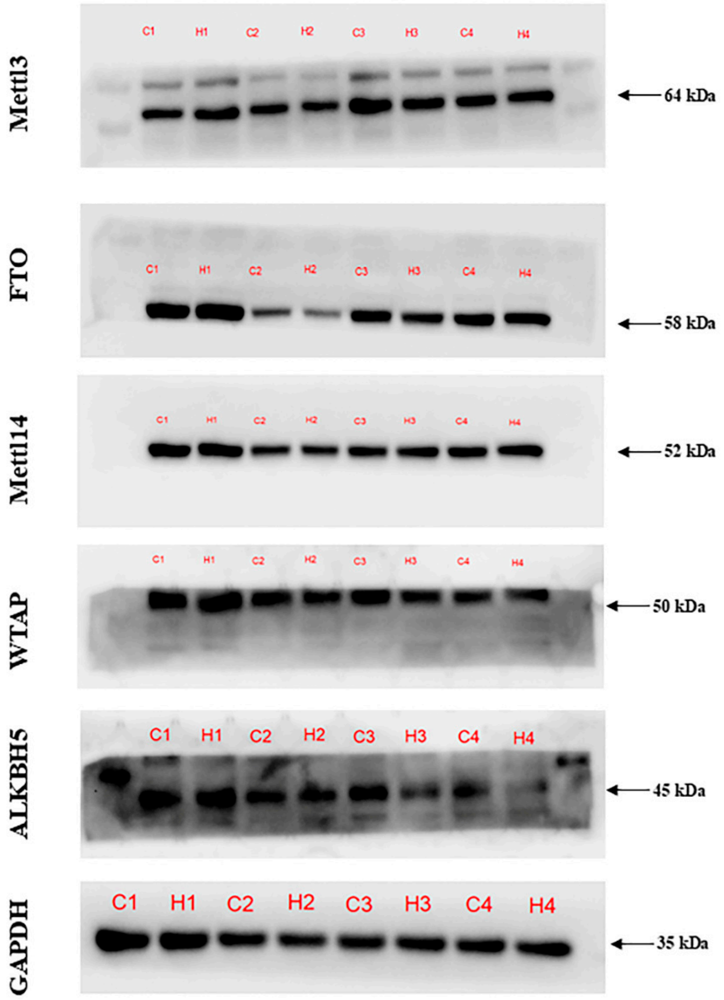

Table S3 Quantitative densitometry data for Western blot analyses

group	GAPDH	METTL3	METTL3/GAPDH	Relative ratio	METTL14	METTL14/GAPDH	Relative ratio	WTAP	WTAP/GAPDH	Relative ratio	FTO	FTO/GAPDH	Relative ratio	ALKBH5	ALKBH5/GAPDH	Relative ratio
Con3	1,745,399.42	2,433,499.66	1.39424	1	2,568,567.00	1.47162	1	2,948,298.33	1.68918	1	1,887,017.44	1.08114	1	1,865,042.22	1.06855	1
HG3	1,982,687.40	1,791,715.09	0.90368	0.64815	2,736,700.00	1.38030	0.93794	2,277,593.03	1.14874	0.68006	1,482,157.82	0.74755	0.6914	1,150,465.25	0.58026	0.54303
Con4	2,063,576.14	1,736,673.90	0.84158	1	2,973,465.00	1.44093	1	2,076,651.83	1.00634	1	2,039,146.83	0.98816	1.0000	1,225,071.41	0.59366	1
HG4	2,383,426.58	1,855,121.18	0.77834	0.92485	3,490,486.00	1.46448	1.01635	2,062,991.63	0.86556	0.86011	2,177,809.38	0.91373	0.9247	617,132.19	0.25893	0.43615
Con6	1,694,329.23	1,638,845.32	0.96725	1	2,705,346.54	1.59671	1	2,313,129.00	1.36522	1	2,262,982.98	1.33562	1.0000	965,122.42	0.56962	1
HG6	2,515,174.70	1,489,463.42	0.59219	0.61224	2,441,273.46	0.97062	0.60789	1,957,675.00	0.77835	0.57013	1,906,966.85	0.75818	0.5677	824,395.11	0.32777	0.57542
Con8	854,963.15	2,459,643.46	2.87690	1	2,410,607.82	2.81955	1	1,775,261.00	2.07642	1	2,423,955.16	2.83516	1.0000	990,158.42	1.15813	1

HG8	1,391,276.57	2,025,022.06	1.45551	0.50593	3,749,463.96	2.69498	0.95582	2,395,604.00	1.72187	0.82925	3,387,536.44	2.43484	0.8588	1,063,667.53	0.76453	0.66014
